# Disordered eating in a tertiary setting: considerations for responding at a university health and counselling service

**DOI:** 10.1186/2050-2974-3-S1-P1

**Published:** 2015-11-23

**Authors:** Maree Burns

**Affiliations:** 1 University Health and Counselling Service, Auckland University, Auckland, New Zealand

## 

Measuring and comparing the prevalence of eating disorders/disordered eating in tertiary settings with the general population is notoriously difficult. However studies indicate that many students in tertiary settings struggle with body dissatisfaction, restriction, purging or a diagnosable eating disorder at any one time. Given that poor body image and dieting can be entrees into eating disorders, this has obvious implications for counselling services in university settings where brief interventions are usually the norm. This paper will consider how to respond to these students focussing on issues of identification, risk, key counselling foci, ‘self-help’ including support group work, collegial relationships within/outside the service, and referral. I will generate discussion about adequately responding to students who may be experiencing and reporting disordered eating for the first time, to a university counselling service.

